# SLR-YOLO: An Improved YOLO-Based Method for Accurate Detection of Potato Leaf Diseases in Complex Field Images

**DOI:** 10.3390/plants15142109

**Published:** 2026-07-08

**Authors:** Tiantian Xu, Lixing Tang, Jingjing Qi, Peng Wang, Guoxiong Zhou

**Affiliations:** Institute of Artificial Intelligence Application, Central South University of Forestry and Technology, Changsha 410004, China; 20233706@csuft.edu.cn (T.X.); 20233702@csuft.edu.cn (L.T.); t20100852@cusft.edu.cn (J.Q.)

**Keywords:** potato leaf disease, object detection, YOLO, LSKA, Mona, precision agriculture

## Abstract

Potato leaf diseases directly reduce yield and quality, and accurate field detection is important for precision plant protection. However, potato disease lesions are often weak in deep semantic representation, easily disturbed by complex field backgrounds, and variable in multi-scale lesion texture. To address these challenges, this study proposes an improved YOLO-based potato leaf disease detection model. The proposed model enhances the detector through three task-oriented modules. Deep Symptom Enhancement is used to strengthen deep disease feature extraction. Lesion Selection Attention based on large separable kernel attention improves the spatial selection of lesion regions. Multi-Scale Refinement Adapter uses a Mona-based C2PSA structure with two stacked Mona adapters to refine multi-scale texture and lesion-boundary information. Experiments were conducted on a potato leaf disease image dataset using mAP50, average recall (AR), parameters, GFLOPs, and FPS as evaluation metrics. The baseline YOLO26s achieved 81.31% mAP50 and 77.85% AR. The proposed SLR-YOLO model achieved 88.92% mAP50 and 83.51% AR, improving mAP50 and AR by 7.61 and 5.66 percentage points, respectively, while maintaining 118.6 FPS. The results show that the proposed framework improves detection accuracy for potato leaf disease images while retaining practical real-time performance.

## 1. Introduction

Potato is one of the most important food and economic crops and is widely planted in many temperate and subtropical regions. Its yield is strongly related to the health condition of the above-ground canopy because the leaf surface is the main organ for photosynthesis and disease symptom expression. In potato production, several diseases first appear on leaves and then gradually influence the whole plant. For example, early blight usually produces brown necrotic spots with concentric texture, while late blight may appear as irregular dark lesions and water-soaked areas under humid conditions. If these symptoms are not detected in time, infection may spread quickly, the effective photosynthetic area decreases, and the final tuber yield and quality are reduced. Therefore, timely and accurate identification of potato leaf disease is an important step in precision plant protection.

In traditional production, disease investigation is mainly completed by growers, technicians, or plant protection experts. This procedure is intuitive and practical, but it also has clear limitations. First, manual inspection is labor-intensive when planting areas are large. Second, diagnosis depends on professional experience, and the same image may be judged differently by different observers. Third, disease symptoms change with infection stage, illumination, leaf age, and environmental humidity, so the diagnostic standard is not always stable. Finally, manual investigation can only sample limited field regions and cannot provide continuous monitoring. With the development of imaging equipment, unmanned platforms, edge computing devices, and deep learning algorithms, image-based plant disease detection has become an effective technical route for intelligent agriculture.

Early image-based disease recognition methods generally extract hand-crafted features, including color histograms, texture descriptors, shape features, and lesion area ratios, and then use classifiers such as support vector machines, random forests, or shallow neural networks to complete category recognition. These methods can work under controlled backgrounds, but their generalization ability is limited. Potato disease images collected in practical scenes often include soil, stems, shadows, overlapping leaves, leaf veins, blur, and illumination changes. Disease lesions may occupy only a small part of the image, and their color may be close to the background or to normal senescent tissue. It is difficult for hand-crafted features to cover all these changes.

Deep learning has greatly improved visual recognition performance. Classification networks can determine the disease type of an image, but they usually assume that the main object fills the image and cannot explicitly output lesion positions. Semantic segmentation networks can obtain pixel-level disease regions, but they require expensive pixel annotation and often have higher computational cost. Object detection networks provide a practical compromise: they can identify disease categories and locate disease regions with bounding boxes. This output is closer to field management because the location of infection can guide targeted spraying, disease severity analysis, and later disease tracking.

Object detection methods can be roughly divided into two categories. Two-stage detectors such as Faster R-CNN first generate candidate regions and then classify and refine them [1]. They usually have strong accuracy but relatively high computational cost. One-stage detectors such as SSD and YOLO directly predict categories and bounding boxes from feature maps [2,3]. Because one-stage detectors are fast and simple to deploy, they have been widely applied to agricultural disease and pest detection. YOLO-series models have developed rapidly, and many variants have improved the backbone, feature pyramid, label assignment, loss function, and detection head [4,5,6]. These improvements provide a strong foundation for real-time crop disease detection.

Many studies have shown that YOLO-based methods are feasible for plant disease detection. Attention mechanisms, multi-scale feature fusion, lightweight backbones, disease-oriented feature aggregation, and improved detection heads have been introduced to improve recognition accuracy under natural backgrounds [7,8,9,10]. These studies indicate that simply applying a general detector is often insufficient. The network structure should be adjusted according to the visual properties of crop disease symptoms.

Potato leaf disease detection exposes three major challenges that directly affect detector design. Deep semantic representation is easily weakened when early lesions are small, blurred, or visually mixed with normal leaf veins. Background interference also causes false responses because soil, stems, shadows, weeds, senescent regions, and overlapping leaves may appear near the disease area and show colors similar to necrotic symptoms. Multi-scale lesion texture further increases detection difficulty because small scattered spots, boundary halos, fragmented edges, and larger necrotic regions may appear in the same image.

These observations motivate a disease-oriented detector in which each structural change corresponds to a specific visual problem. This study constructs an improved YOLO-based detector for potato leaf disease images. The goal is not to simply stack modules, but to connect each network modification with deep semantic enhancement, background interference suppression, and multi-scale lesion texture refinement.

The proposed method improves the detector from three aspects. Deep Symptom Enhancement is introduced into the deep feature extraction stage to strengthen contextual semantic information. Lesion Selection Attention is introduced to enhance spatial feature selection for lesion regions under complex field interference. A Multi-Scale Refinement Adapter with two Mona adapters is used after SPPF to refine multi-scale disease features. Mona can perform multi-scale depthwise convolution and lightweight adapter mapping, which are suitable for disease symptoms with different lesion sizes.

The contributions of this study are summarized as follows:

1. A potato leaf disease detection framework based on an improved YOLO26s model is designed. The framework is organized around deep semantic enhancement, background interference suppression, and multi-scale lesion texture refinement.
2. Deep Symptom Enhancement is introduced into the deep feature extraction stage. This module improves contextual feature extraction and helps the network preserve discriminative information for weak and ambiguous symptoms.
3. Lesion Selection Attention is used to enhance spatial feature selection for irregularly distributed lesion regions, reducing interference from soil, shadows, and healthy leaf texture.
4. A Multi-Scale Refinement Adapter is designed for multi-scale disease feature refinement. It uses a two-branch structure and multi-kernel depthwise convolution to enhance lesion texture, edge, and local semantic information.
5. A complete experimental scheme is provided, including training settings, baseline comparison, module effectiveness analysis, ablation experiments, complexity analysis, and qualitative visualization.

## 2. Related Work

### 2.1. Deep Learning-Based Plant Disease Detection

Image-based plant disease analysis has developed from hand-crafted feature recognition to deep learning visual modeling. Traditional pipelines usually describe disease symptoms through color, texture, shape, and lesion area features, and then use shallow classifiers to distinguish disease categories. These methods are interpretable under controlled imaging conditions, but their feature design is sensitive to illumination, background, leaf age, and lesion morphology. Deep convolutional networks reduced this dependence by learning feature representations directly from leaf images, and large disease image datasets further promoted data-driven disease recognition [11,12].

Representative methods have improved plant disease recognition from several directions. CNN classification models achieved strong accuracy on leaf image datasets, while transfer learning reduced the training difficulty when available disease samples were limited [11,12,13]. Real scene datasets moved the task from clean background classification toward disease understanding under natural backgrounds [14]. Region-based convolutional detectors introduced lesion localization ability, and segmentation benchmarks showed that pixel-level masks can describe lesion boundaries more precisely [15,16]. Transformer-based classification also showed that global dependency modeling can help represent complex symptom patterns, although this usually increases computational cost [17].

These studies promoted plant disease recognition, but several limitations remain. Classification methods usually lack explicit lesion positions, segmentation methods require expensive pixel-level annotation, and two stage detection methods may be less efficient for real time field monitoring. More importantly, weak symptoms and ambiguous lesions may be gradually weakened in deep features when a general backbone is directly used. This problem is obvious in potato leaf disease detection because early lesions and blurred necrotic regions often need stronger semantic context for reliable discrimination.

These observations motivate a detector that can preserve deep disease semantics while keeping the speed advantage of a one-stage framework. SLR-YOLO therefore introduces Deep Symptom Enhancement (DSE) in the deep backbone stage. This module is designed to strengthen the semantic representation of weak and ambiguous potato leaf disease symptoms and to provide more discriminative high-level features for later localization.

### 2.2. YOLO-Based Detection Methods in Agricultural Images

YOLO-based detectors have become widely used in agricultural vision because they combine object localization and category prediction in a single-stage framework. In practical agricultural scenarios, the detector must process large numbers of images or video frames and provide timely responses for field management. Compared with many two-stage detectors, YOLO models are easier to deploy and usually provide better inference speed. This property makes them suitable for detecting crop diseases, pests, fruits, weeds, grains, and other agricultural objects in natural scenes [3,4,5].

Existing agricultural YOLO studies often improve the detector through lightweight backbones, multi-scale detection, modified heads, and task-specific training strategies. MobileNet style backbones and multi-scale parallel detection designs improve disease detection efficiency and lesion scale adaptability under natural environments [18,19]. Modified YOLOv3 frameworks have also been applied to greenhouse object detection, where occlusion and color similarity increase detection difficulty [20]. Improved YOLOv8 models further show that efficient heads, deep supervision, and dynamic upsampling can support lightweight pest detection [21]. Comparative studies involving SSD, YOLO, Transformer-based detectors, and small object-oriented YOLO designs confirm that agricultural targets usually require a balance among speed, recall, and scale-sensitive localization [22,23,24].

Although these studies improve agricultural detection performance, complex field backgrounds still cause false responses. Soil, stems, shadows, dead leaves, and overlapping leaf regions may have colors or textures similar to necrotic symptoms. A detector that only enhances the backbone or detection head may still pass background interference into the feature fusion process. This limitation is especially important for potato leaf disease detection because lesions often appear near leaf edges or are mixed with natural leaf texture.

This background interference motivates a more selective feature response before later fusion. SLR-YOLO therefore introduces Lesion Selection Attention (LSA). This module strengthens lesion region selection and suppresses background responses that are not useful for disease localization, which directly supports potato lesion detection in complex field images.

### 2.3. Feature Enhancement and Attention Mechanisms for Disease Lesion Detection

Plant disease detection differs from general object detection because lesion regions are often small, irregular, visually similar across classes, and mixed with complex leaf texture. Feature enhancement and attention mechanisms have therefore become important strategies for disease lesion detection. Feature pyramid networks and attention modules provide useful tools for combining high-level semantics with low-level details and for assigning stronger responses to informative regions [25,26]. In disease images, these mechanisms are usually used to improve lesion representation, suppress irrelevant background, or fuse multi-scale symptom information.

Representative disease-oriented detectors have introduced more specific enhancement designs. Perceptual adaptive convolution, location reinforcement attention, and proximity feature aggregation improve disease feature perception and feature fusion [8]. Prior knowledge attention and multi-scale feature fusion guide disease target detection with symptom related visual cues [9]. Transformer assisted backbones, dynamic activation, and improved bidirectional feature pyramids improve small lesion localization and reduce false detection under complex scenes [10]. Multi-scale parallel convolution and recent YOLO or DETR variants further show that different receptive fields, attention modules, and adaptive feature fusion can improve plant disease detection under scale variation and background disturbance [27,28,29,30].

These studies confirm that attention, multi-scale modeling, and feature fusion are effective for disease lesion detection. However, many methods focus on one aspect of the problem, such as background suppression, multi-scale fusion, or lightweight detection. Potato leaf disease images require these abilities to work together because weak symptoms, field interference, and lesion scale variation appear simultaneously. Lesions may be small and scattered at an early stage, while severe symptoms may form larger and irregular necrotic areas. A single receptive field is therefore insufficient for stable symptom representation.

This multi-scale texture problem motivates a refinement module after spatial pyramid pooling. SLR-YOLO uses Multi-Scale Refinement Adapter (MSRA) after SPPF to refine lesion texture, boundary, and local semantic information. Together with DSE and LSA, MSRA forms a task-oriented refinement pipeline for complex potato leaf disease images.

## 3. Materials and Methods

### 3.1. Baseline Network and Model Motivation

**Figure 1:**
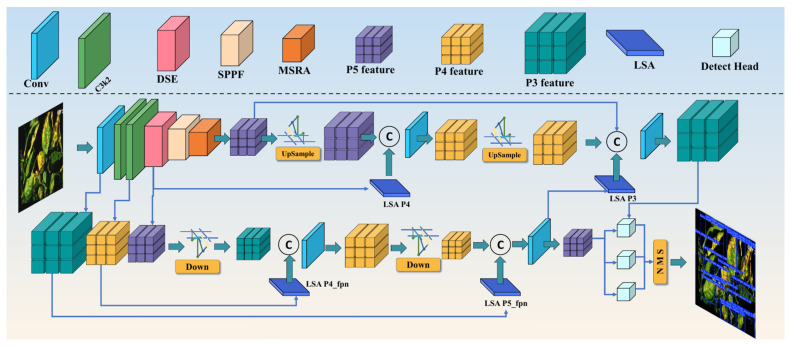
Overall architecture of SLR-YOLO for potato leaf disease detection in complex field images.

The baseline model is YOLO26s (Figure 1). It is selected as the starting detector because it provides a strong single-stage detection pipeline, fast inference, and a clear backbone, neck, and head structure. These properties are important for potato leaf disease monitoring, where the model must locate lesion regions while keeping enough speed for field use. The input image is denoted as $I\in R^{3\times H\times W}$, and its annotations are converted to the YOLO format. Each target is represented by a category label and a normalized box b=(x,y,w,h). This setting allows the detector to learn both symptom category and lesion position.

The baseline backbone extracts hierarchical visual features through convolution and C3k2 blocks. Shallow stages preserve edges, color transitions, and local texture, while deeper stages encode stronger semantic information about disease categories. The neck receives features from different backbone stages and fuses them through upsampling, concatenation, and downsampling operations. This design allows the detector to use both fine spatial details and high-level semantics. The detection head then predicts lesion bounding boxes and disease class probabilities at multiple scales, which is suitable for disease regions with different sizes.

YOLO26s is a reasonable baseline for this study because it provides a practical balance between accuracy, model complexity, and inference speed. Potato leaf disease detection needs this balance because field monitoring often requires timely localization rather than offline image classification only. The baseline also has a modular structure, so backbone enhancement, attention selection, and post SPPF refinement can be inserted without changing the overall one stage detection pipeline.

The baseline is efficient, yet potato disease images expose three weaknesses. Deep layers may weaken small and ambiguous symptoms when lesion texture is subtle. Field background may generate lesion-like responses when soil, shadows, or senescent leaf regions have similar color patterns. The feature after spatial pyramid pooling still needs stronger texture refinement because small spots, lesion boundaries, and larger necrotic areas may appear in the same image. The proposed model is therefore named SLR-YOLO, namely Symptom Lesion Refinement YOLO. It keeps the efficient YOLO26s pipeline and introduces Deep Symptom Enhancement, Lesion Selection Attention, and Multi-Scale Refinement Adapter.

### 3.2. Deep Symptom Enhancement

Deep Symptom Enhancement is introduced at the fourth backbone stage, where high-level disease semantics have formed and the feature map still preserves useful spatial cues. The input of this stage is denoted as $X_{d}\in R^{C\times H_{d}\times W_{d}}$. The stage begins with a channel projection that prepares two information paths:

(1) $$U=\phi_{1\times 1}\left(X_{d}\right),\;\left[U_{a},U_{b}\right]=\mathrm{Split}\left(U\right),\;C_{a}+C_{b}=C.$$

The input $X_{d}$ comes from the fourth backbone stage. The projection $\phi_{1\times 1}$ adjusts its channel representation and produces *U*. The split operation separates *U* into the enhancement branch $U_{a}$ and the preserved branch $U_{b}$.

The branch $U_{b}$ preserves stable backbone information. The branch $U_{a}$ is passed through efficient visual mixing and channel projection:

(2) $$S_{t}=M_{\theta }\left(U_{a}\right)={\mathrm{Mix}}_{\theta }\left(U_{a}\right)+U_{a},\;{\tilde{S}}_{t}=\phi_{1\times 1}\left(S_{t}\right).$$

The operator $M_{\theta }$ is the efficient visual mixing function with learnable parameters θ. Its output $S_{t}$ contains wider spatial symptom information, while ${\tilde{S}}_{t}$ keeps the response suitable for attention computation.

Because weak potato symptoms may be hidden in leaf veins and normal texture, a position-sensitive attention response is generated from the deep feature:

(3) $$\begin{array}{cc} T & =\mathrm{Flat}\left({\tilde{S}}_{t}\right)\in R^{C_{q}\times N},\;N=H_{d}W_{d},\\ Q & =W_{q}T,\;K=W_{k}T,\;V=W_{v}T,\\ A_{d} & =\mathrm{Softmax}\left(\frac{Q^{T}K}{\sqrt{C_{q}}}\right),\;S_{a}=\mathrm{Reshape}\left(VA_{d}^{T}\right). \end{array}$$

The feature ${\tilde{S}}_{t}$ is flattened into *T* so that spatial positions can be compared in a matrix form. The features *Q*, *K*, and *V* serve as query, key, and value representations. The matrix $A_{d}$ gives the deep attention weights, and $S_{a}$ is the attention enhanced symptom response after reshaping.

The attended feature is further refined by the C3k-style local convolution and then fused with the preserved branch:

(4) $$S_{c}=C_{3k}\left(S_{a}\right),\;S_{f}={\mathrm{Concat}}_{c}\left(S_{c},U_{b}\right),\;X_{d}^{\mathrm{DSE}}=\phi_{1\times 1}\left(S_{f}\right).$$

The operation $C_{3k}$ represents the stacked local convolution in the enhanced block. The feature $S_{f}$ combines the enhanced branch and preserved branch, and $X_{d}^{\mathrm{DSE}}$ is the final output of DSE.

This output is sent to the subsequent SPPF stage:

(5) $$X_{s}=S_{\mathrm{SPPF}}\left(X_{d}^{\mathrm{DSE}}\right)=\phi_{s}\left({\mathrm{Concat}}_{c}\left(X_{d}^{\mathrm{DSE}},P_{1},P_{2},P_{3}\right)\right),\;P_{r}={\mathrm{MaxPool}}^{r}\left(X_{d}^{\mathrm{DSE}}\right).$$

**Figure 2:**
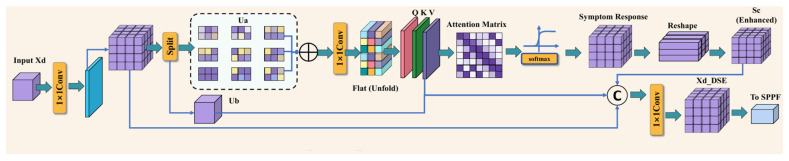
Deep Symptom Enhancement module.

The function $S_{\mathrm{SPPF}}$ denotes the spatial pyramid pooling fast operation, and its output $X_{s}$ is passed to the later refinement stage. Through these operations, DSE strengthens deep symptom semantics before the neck receives high-level information. The module therefore addresses the first difficulty, where small lesions and weak symptoms may be weakened during deep feature extraction. Figure 2 shows the structure of the DSE module.

### 3.3. Lesion Selection Attention

Lesion Selection Attention is used to reduce field interference before disease features are fused at later stages. Its input is a feature map $X_{l}\in R^{C\times H_{l}\times W_{l}}$ that contains lesion responses and background responses. The module first obtains local lesion evidence and broader spatial context:

(6) $$Z_{l}=\phi_{1\times 1}\left(X_{l}\right),\;Z_{s}={\mathrm{DWConv}}_{k_{s}}\left(Z_{l}\right),\;Z_{g}={\mathrm{DWConv}}_{k_{g},d_{g}}\left(Z_{s}\right).$$

The feature $Z_{l}$ is the compressed representation of $X_{l}$. The feature $Z_{s}$ describes local lesion evidence, while $Z_{g}$ uses a larger kernel $k_{g}$ and dilation rate $d_{g}$ to capture broader contextual evidence.

The local and large context features are fused, and a channel selection vector is generated from the fused representation,

(7) $$Z_{c}=Z_{s}+Z_{g},\;z_{\mathrm{avg}}=\mathrm{GAP}\left(Z_{c}\right),\;g_{c}=\sigma \left(W_{2}\delta \left(W_{1}z_{\mathrm{avg}}\right)\right),\;{\bar{Z}}_{c}=g_{c}⊙Z_{c}.$$

The fused feature $Z_{c}$ combines local cues and wider spatial context. Global average pooling summarizes it into $z_{\mathrm{avg}}$, and the gating function produces $g_{c}$ for channel selection. The selected feature ${\bar{Z}}_{c}$ keeps channels that are more useful for lesion discrimination.

Spatial lesion evidence is computed from average and maximum maps, and these two maps are converted into a lesion selection mask,

(8) $$\begin{array}{cc} M_{\mathrm{avg}} & ={\mathrm{Mean}}_{c}\left({\bar{Z}}_{c}\right),\;M_{\max}={\mathrm{Max}}_{c}\left({\bar{Z}}_{c}\right),\\ A_{l} & =\sigma \left(\phi_{k\times k}\left({\mathrm{Concat}}_{c}\left(M_{\mathrm{avg}},M_{\max}\right)\right)\right),\;A_{l}\in \left[0,1\right]. \end{array}$$

The maps $M_{\mathrm{avg}}$ and $M_{\max}$ describe average and strongest spatial responses. The convolution $\phi_{k\times k}$ converts these summaries into the lesion selection mask $A_{l}$.

The selected feature is then passed to the neck,

(9) $$X_{l}^{\mathrm{LSA}}=X_{l}⊙A_{l}+X_{l},\;F_{l}^{\mathrm{LSA}}=N_{l}\left(X_{l}^{\mathrm{LSA}},F_{l+1}\right)=\psi_{l}\left({\mathrm{Concat}}_{c}\left(X_{l}^{\mathrm{LSA}},\mathrm{Up}\left(F_{l+1}\right)\right)\right).$$

**Figure 3:**
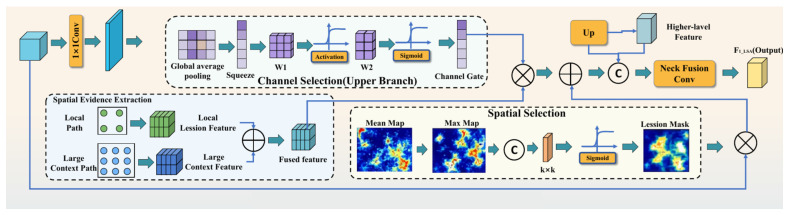
Lesion Selection Attention module.

The output $X_{l}^{\mathrm{LSA}}$ keeps the original information through the residual term $X_{l}$, while the mask $A_{l}$ highlights lesion related positions. The fusion operation $N_{l}$ combines this selected feature with the higher scale feature $F_{l+1}$. LSA therefore performs a selection process before feature fusion. The previous step supplies lesion and background responses, while this step highlights lesion related spatial positions. The next neck operation receives a cleaner feature map, which reduces false detections caused by soil, shadows, and healthy leaf texture. Figure 3 shows the structure of the LSA module.

### 3.4. Multi-Scale Refinement Adapter

Multi-Scale Refinement Adapter is placed after SPPF, where the feature already contains high-level semantics but still needs lesion texture refinement. Its input is denoted as $X_{s}\in R^{C\times H_{s}\times W_{s}}$. The adapter follows the C2PSA style two branch structure used in the implementation. A 1×1 convolution first generates two branches,

(10) $$\left[R_{a},R_{b}\right]=\mathrm{Split}\left(\phi_{1\times 1}^{r}\left(X_{s}\right)\right),\;{\bar{R}}_{a}=\phi_{1\times 1}\left(R_{a}\right).$$

The input $X_{s}$ is the SPPF output feature. After the 1×1 projection, the feature is divided into the stable branch $R_{a}$ and the refinement branch $R_{b}$. The projected branch ${\bar{R}}_{a}$ keeps stable high-level semantic information from $R_{a}$. The right branch is refined by stacked Mona adapters. Each adapter begins with normalization and projection into an adapter space,

(11) $${\hat{R}}_{b}=\mathrm{LN}\left(R_{b}\right)⊙\gamma +R_{b}⊙\gamma_{x},\;P_{1}=W_{1}{\hat{R}}_{b}.$$

Layer normalization LN stabilizes the refinement branch. The learnable factors γ and $\gamma_{x}$ balance normalized information and residual information, and $W_{1}$ maps the result into the adapter feature $P_{1}$.

The adapter then extracts lesion texture at three receptive field sizes and fuses them with the identity feature:

(12) $$\begin{array}{cc} D_{3} & ={\mathrm{DWConv}}_{3\times 3}\left(P_{1}\right),\;D_{5}={\mathrm{DWConv}}_{5\times 5}\left(P_{1}\right),\\ D_{7} & ={\mathrm{DWConv}}_{7\times 7}\left(P_{1}\right),\;D_{m}=\frac{D_{3}+D_{5}+D_{7}}{3}+P_{1}. \end{array}$$

The responses $D_{3}$, $D_{5}$, and $D_{7}$ describe fine spots, middle-scale texture, and wider necrotic context. The fused feature $D_{m}$ combines these scale responses and keeps the original adapter representation through $P_{1}$.

The fused response is projected, activated, and mapped back to the original branch dimension:

(13) $$\begin{array}{cc} D_{p} & =W_{m}D_{m},\;D_{o}=D_{m}+D_{p},\\ D_{a} & =\mathrm{Dropout}(\mathrm{GELU}\left(D_{o}\right)),\\ P_{2} & =W_{2}D_{a},\;R_{b}^{\prime }=R_{b}+P_{2}. \end{array}$$

The projection $D_{p}$ mixes channel information after multi-scale depthwise convolution. The output $D_{o}$ keeps residual information, $D_{a}$ is the activated and regularized feature, and $R_{b}^{\prime }$ is the output of one Mona adapter.

After *n* adapters, the refined branch can be written as

(14) $$R_{b}^{\left(0\right)}=R_{b},\;R_{b}^{\left(j\right)}=A_{j}\left(R_{b}^{\left(j-1\right)}\right),\;j=1,\ldots ,n.$$

The symbol $A_{j}$ denotes the *j*th Mona adapter, and *n* is the number of stacked adapters. The branch $R_{b}^{\left(n\right)}$ is the refined result after repeated adapter processing.

The stable branch and refined branch are finally concatenated and sent to the neck:

(15) $$R_{f}={\mathrm{Concat}}_{c}\left({\bar{R}}_{a},R_{b}^{\left(n\right)}\right),\;X_{s}^{\mathrm{MSRA}}=\phi_{1\times 1}\left(R_{f}\right),\;F_{5}=N_{5}\left(X_{s}^{\mathrm{MSRA}}\right)=\psi_{5}\left(X_{s}^{\mathrm{MSRA}}\right).$$

**Figure 4:**
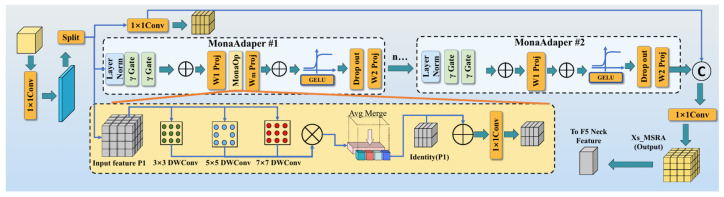
Multi Scale Refinement Adapter module.

The feature $R_{f}$ combines stable semantic information from ${\bar{R}}_{a}$ and refined multi-scale lesion information from $R_{b}^{\left(n\right)}$. The final MSRA output $X_{s}^{\mathrm{MSRA}}$ is produced by the fusion convolution, and $F_{5}$ is the highest-level neck feature. MSRA therefore receives the SPPF output, refines lesion texture through multi-scale depthwise convolution, and transfers richer high-level information to the neck. The module addresses the third difficulty, where small spots, lesion boundaries, necrotic texture, and larger infected regions need to be represented in a unified feature space. Figure 4 shows the structure of the MSRA module.

### 3.5. Training Settings

All models were trained under the same basic training strategy to ensure fair comparison. The optimizer, learning rate schedule, epoch number, batch size, input size, and augmentation strategy were kept consistent across the baseline and improved models (Figure 1). The training configuration is shown in Table 1.

### 3.6. Evaluation Metrics

Mean average precision at an IoU threshold of 0.50 (mAP50) and average recall (AR) are used to evaluate detection accuracy. mAP50 reflects the mean detection precision of all disease categories under a 0.50 IoU threshold. AR measures the proportion of ground-truth objects successfully recalled by the detector and is important for disease monitoring because missed disease regions may delay field management.

The computational complexity is evaluated by parameters, FLOPs, and FPS. Parameters indicate model capacity. FLOPs reflect theoretical computation. FPS measures inference speed and is important for practical deployment. For agricultural applications, a model should not only obtain high accuracy but also maintain reasonable speed.

## 4. Results

### 4.1. Experimental Arrangement

The result section is organized to verify whether the proposed method solves the problems described above. General detection performance is reported against the baseline. The proposed model is then compared with representative detectors. The effects of attention mechanisms, backbone enhancement positions, Mona adapter depth, and module combinations are analyzed. Visualization results are finally used to observe missed detections, false detections, and localization quality.

### 4.2. Overall Performance of the Proposed Model

The overall performance of the proposed model was first compared with the baseline YOLO26s. This comparison directly shows whether the disease-oriented modifications improve the detector. Table 2 reports the main accuracy and efficiency metrics.

**Figure 5:**
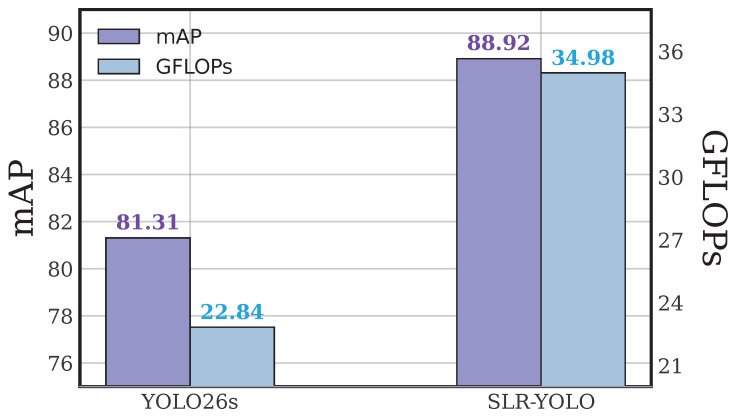
Overall performance comparison between the baseline and proposed model.

As shown in Table 2, the proposed model achieved 88.92% mAP50 and 83.51% AR. Figure 5 further visualizes the same comparison and shows the accuracy gain together with the speed change. Compared with the YOLO26s baseline, mAP50 increased by 7.61 percentage points and AR increased by 5.66 percentage points. This indicates that the improved model can detect more potato disease regions while maintaining higher overall detection accuracy. The parameter number increased slightly from 10.010 M to 10.824 M, while GFLOPs increased from 22.84 G to 34.98 G. Although FPS decreased from 156.9 to 118.6, the proposed model still satisfies real-time detection requirements.

The performance trade-off is acceptable for potato leaf disease monitoring. In practical field applications, missed disease regions may lead to delayed control measures. Therefore, the improvement in AR is particularly valuable. The proposed model uses additional computation to enhance disease feature extraction and multi-scale refinement, but its inference speed remains high enough for camera-based or workstation-based disease detection.

### 4.3. Comparison with Baseline and Existing Detectors

To verify the effectiveness of the proposed model, it was compared with representative one-stage and transformer-based detectors, including YOLOv5S, YOLOv8S, YOLOv11S, YOLO26s, RTDETR_L, SPPELAN, and EfficientDet-D0. All models were trained and tested on the same potato leaf disease dataset. The comparison results are shown in Table 3.

**Figure 6:**
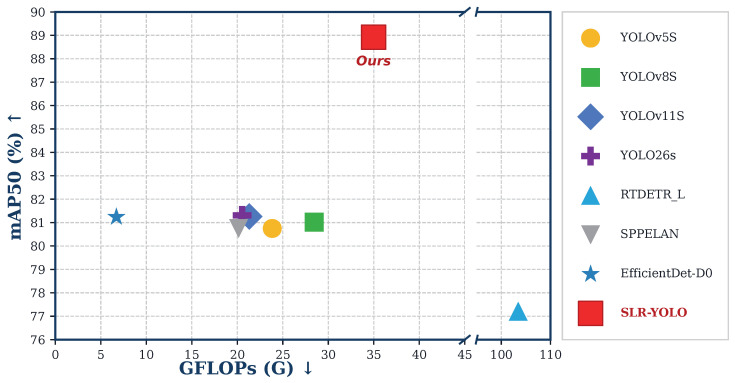
Accuracy efficiency landscape of representative detectors.

Different detectors show different performance characteristics. Figure 6 presents the accuracy and computational efficiency distribution of the compared detectors. YOLOv5S and YOLOv8S achieved high FPS, but their mAP50 and AR were lower than those of the proposed model. YOLO26s reached 81.31% mAP50 and 77.85% AR, but its accuracy was still lower than that of the proposed model. RTDETR_L had the largest parameter number and GFLOPs, but its mAP50 and FPS were both lower, indicating that a heavier detector is not necessarily more suitable for this potato leaf disease task.

Compared with all tested detectors, the proposed model achieved the highest mAP50 and AR. Although its FPS was lower than that of the smallest YOLO models, it still reached 118.6 FPS, which is sufficient for real-time detection. This result shows that the proposed disease-oriented improvements provide a better accuracy-speed balance for potato leaf disease images.

### 4.4. Comparison of Attention Mechanisms

To determine a suitable attention mechanism for potato leaf disease detection, several attention modules were compared under the same baseline. The results are shown in Table 4. This experiment evaluates whether the detector benefits more from channel attention, spatial attention, or large-kernel spatial selective attention.

**Figure 7:**
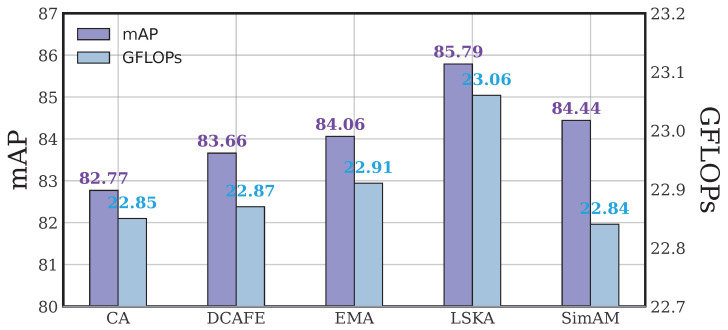
Performance gain of different attention mechanisms.

As shown in Table 4, all tested attention mechanisms improved the baseline to different degrees. Figure 7 compares the mAP50 gain and AR of these attention variants. Among them, LSKA obtained the best performance, with 85.79% mAP50 and 80.44% AR. Compared with the baseline, LSKA improved mAP50 by 4.48 percentage points and AR by 2.59 percentage points. Its parameter and computational increases were small. The measured FPS in this independent attention experiment reached 186.6, which shows that the inserted attention branch did not become the main inference bottleneck under the tested runtime setting. This result indicates that large separable kernel spatial modeling is effective for potato leaf disease features, which often have irregular spatial distribution and variable lesion scale.

### 4.5. Backbone Enhancement Position Experiment

The backbone enhancement position determines where the model strengthens feature extraction. To identify the most suitable position, several backbone enhancement schemes were tested. The results are shown in Table 5.

**Figure 8:**
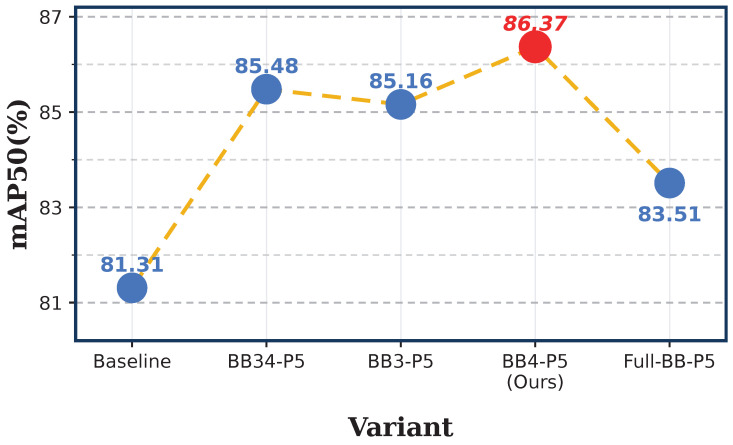
Effect of different backbone enhancement positions.

The DSE variant placed at BB4-P5 achieved the best performance among the backbone enhancement variants, with 86.37% mAP50 and 80.73% AR. Figure 8 shows that this position gives the strongest accuracy while avoiding the speed loss of Full-BB-P5. Compared with the baseline, mAP50 and AR increased by 5.06 and 2.88 percentage points, respectively. Although BB34-P5 and BB3-P5 also improved mAP50, their AR values were lower than that of BB4-P5. Full-BB-P5 introduced the largest computation and reduced FPS to 62.8, but its accuracy was not the best. This indicates that enhancing all backbone stages may introduce redundant features or excessive complexity, while the BB4-P5 placement provides a better balance.

### 4.6. Effectiveness of Mona Depth

The Mona module refines multi-scale disease features after SPPF. Different numbers of Mona adapters were tested to determine the best depth. The results are shown in Table 6.

**Figure 9:**
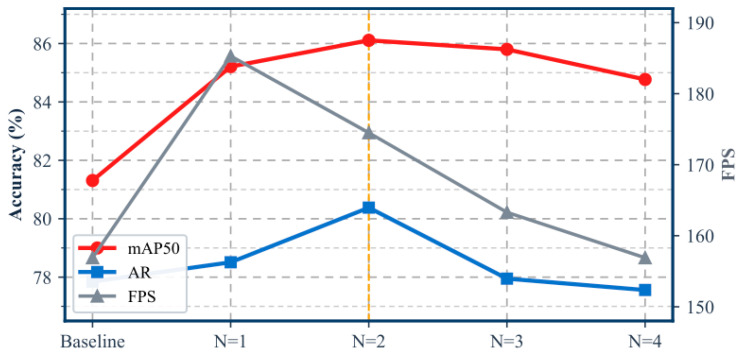
Effect of Mona adapter depth on detection performance.

Mona N = 2 obtained the best result, with 86.11% mAP50 and 80.38% AR. Figure 9 shows that the accuracy increases from N = 1 to N = 2 and then decreases when the adapter depth continues to grow. Increasing the number of Mona adapters from 1 to 2 improved both mAP50 and AR, indicating that a moderate depth helps the model refine multi-scale lesion texture. However, when the depth increased to 3 or 4, accuracy decreased. This suggests that excessive adapter stacking may introduce redundant transformation or overfit the training data. Therefore, Mona N = 2 was selected for the final combination.

### 4.7. Ablation Experiment

An ablation experiment was conducted to evaluate the combined contribution of BB4, LSKA, and Mona N = 2. The results are shown in Table 7.

**Figure 10:**
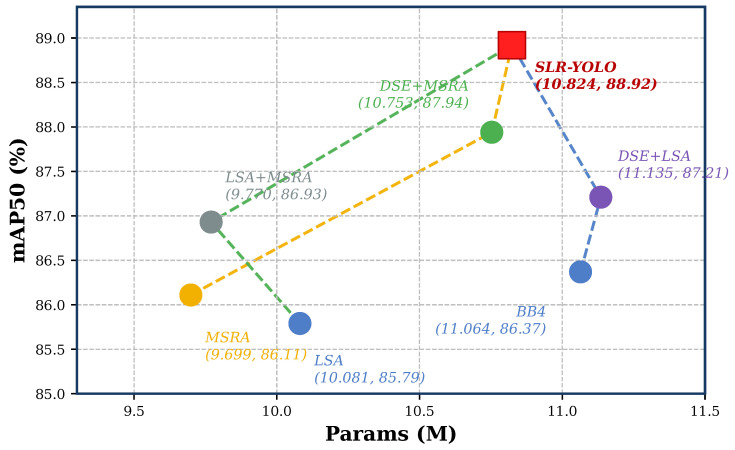
Performance and parameter trade-off of ablation variants.

The ablation experiment verifies the cumulative effect of the proposed modules. Figure 10 visualizes the mAP50 and parameter trade-off of each single module and module combination. When used independently, DSE, LSA, and MSRA all improved the baseline substantially. DSE achieved 86.37% mAP50 and 80.73% AR, LSA achieved 85.79% mAP50 and 80.44% AR, and MSRA achieved 86.11% mAP50 and 80.38% AR. These results show that backbone enhancement, spatial attention, and multi-scale feature refinement are all effective for potato leaf disease detection.

The combination results further show complementarity among the modules. The model using DSE and MSRA achieved 87.94% mAP50 and 82.66% AR, outperforming either module alone. The model using DSE and LSA achieved 87.21% mAP50 and 81.94% AR, while the model using LSA and MSRA achieved 86.93% mAP50 and 81.28% AR. The final model using DSE, LSA, and MSRA achieved the best overall performance, with 88.92% mAP50 and 83.51% AR. Compared with the YOLO26s baseline, the final model improved mAP50 by 7.61 percentage points and AR by 5.66 percentage points.

### 4.8. Complexity and Speed Analysis

In addition to detection accuracy, model complexity and inference speed are important for agricultural applications. Table 8 compares the parameters, GFLOPs, and FPS of the main tested detectors.

**Figure 11:**
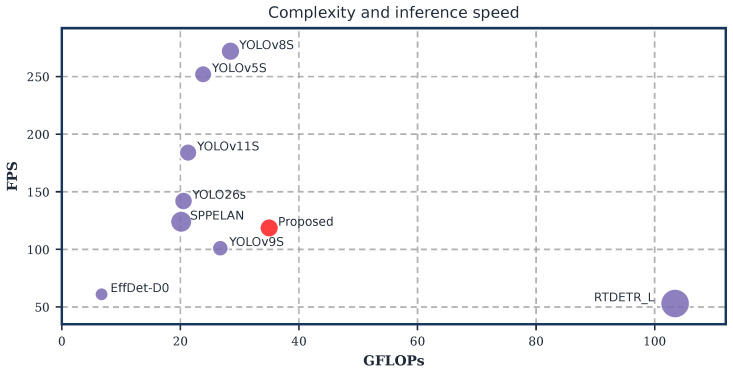
Complexity and speed distribution of representative detectors.

Figure 11 shows the relationship between computation and inference speed for the representative detectors. The proposed model has 10.824 M parameters, which is close to YOLOv8S and slightly higher than YOLO26s. Its GFLOPs are higher than those of the baseline because DSE, LSA, and MSRA increase feature modeling cost. Nevertheless, the proposed model still reaches 118.6 FPS. Compared with RTDETR_L, the proposed model is much lighter and faster while obtaining higher detection accuracy. Compared with EfficientDet-D0, the proposed model has higher computational cost but much better accuracy and speed. Therefore, the proposed model provides a practical balance between accuracy and efficiency.

### 4.9. Visualization of Detection Results

**Figure 12:**
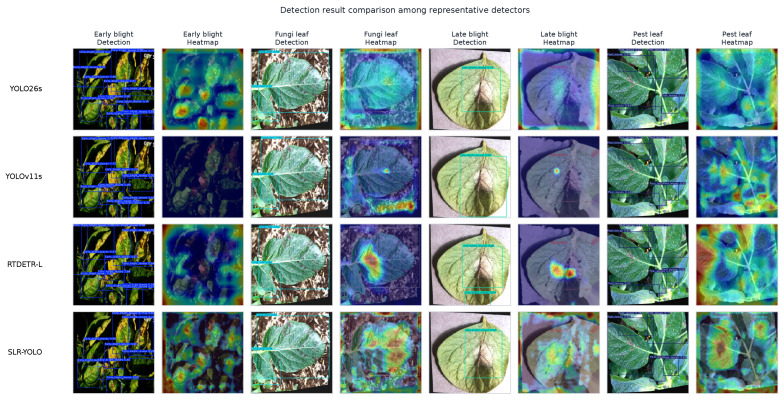
Visual comparison of detection results and model-specific attention heatmaps among representative detectors.

The visual detection results further show how the proposed model behaves in complex potato leaf images. Figure 12 compares representative detectors and their model-specific attention heatmaps on the same selected disease samples. The heatmaps are used as qualitative visualization evidence rather than as independent accuracy metrics. They were generated from the corresponding trained model under the same selected input samples, and each heatmap is paired with the detection result of the same model. YOLO26s provides a useful baseline, but some dense or weak lesions still receive incomplete responses. YOLOv11s and RTDETR_L show different localization and response patterns under leaf overlap and complex texture. SLR-YOLO produces more stable responses across early blight, fungi leaf, late blight, and pest leaf samples.

**Figure 13:**
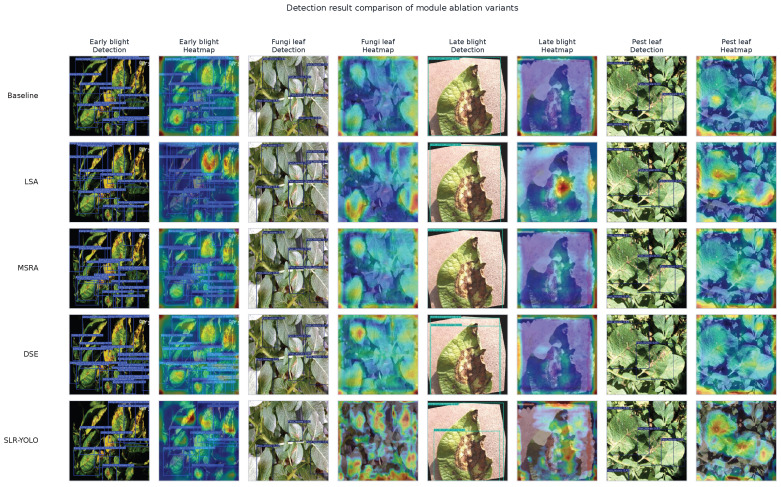
Visual comparison of detection results and model-specific attention heatmaps for the ablation variants.

The module level visual comparison is shown in Figure 13. Each ablation variant is displayed with its own detection result and attention heatmap, and the paired heatmap uses the same image as the corresponding detection result. The baseline model can locate many obvious lesions, while several small or texture mixed regions remain difficult. LSA improves the spatial selection of disease regions. MSRA enhances lesion texture responses after multi-scale refinement. DSE strengthens deep symptom semantics and helps the detector maintain responses on ambiguous disease regions. The final SLR-YOLO combines these effects and gives more complete lesion localization under different disease appearances.

## 5. Discussion

The proposed method is designed according to the visual characteristics of potato leaf diseases. Compared with a generic YOLO detector, the improved model has stronger disease-specific feature extraction capability. DSE enhances deep backbone representation, which is helpful when disease symptoms are weak or visually mixed with normal leaf texture. LSA improves spatial attention to lesion regions with variable shapes and distributions. MSRA introduces multi-scale local modeling, allowing the network to describe lesion spots, halos, necrotic edges, and larger infected areas in a unified structure.

This design follows a disease-oriented principle in which the network structure should correspond to actual detection challenges rather than simply stack general modules. In potato leaf disease images, the main challenges are deep semantic weakening, background interference, and multi-scale lesion texture variation. Therefore, the proposed model adapts the deep backbone stage, the spatial attention stage, and the post SPPF refinement stage simultaneously.

Compared with methods that only replace the backbone with a lightweight network, the proposed model pays more attention to disease feature quality. Lightweight networks may improve speed, but they may also lose subtle texture information. Potato disease lesions require the model to preserve small spots, color transition, and boundary details. Therefore, the proposed method chooses a moderate enhancement strategy rather than aggressive compression. The experimental results show that the accuracy gain is clear, while the final model still maintains 118.6 FPS.

The three improvements have different roles. DSE is responsible for extracting more complete disease representations from deep features. LSA is responsible for assigning stronger spatial responses to useful lesion regions. MSRA is responsible for refining multi-scale lesion texture and semantic information. The ablation results show that each module improves the baseline, and the final combination achieves the highest mAP50 and AR. This confirms that backbone enhancement, attention-based feature selection, and multi-scale texture refinement are complementary.

The dataset also plays an important role. If the training images include many clear laboratory samples but few natural field samples, the model may perform well on the test set but less robustly in real scenes. If the dataset contains severe class imbalance, the detector may prefer categories with more annotations. This problem is common in disease detection because some diseases produce many small spots while others produce fewer large lesions. Therefore, future work should consider balancing both image numbers and annotation numbers.

Another issue is disease stage. Potato disease detection is not only a category recognition problem but also a severity and development-stage problem. Early warning requires detecting weak symptoms before large lesions appear. However, early symptoms may be visually similar to noise, water droplets, or natural texture. A pure RGB detection model may be insufficient in some cases. In the future, multi-modal information such as hyperspectral images, environmental sensors, or temporal image sequences can be combined with the detector to improve early disease warning.

Deployment should also be considered. The proposed model improves accuracy, but its GFLOPs increase from 22.84 G to 34.98 G compared with the baseline. If the model is deployed on mobile or edge devices, pruning, quantization, or knowledge distillation may be necessary. In addition, the current experimental data mainly report mAP50, AR, complexity, and FPS. Future experiments should further provide per-class AP, mAP50–95, confusion matrices, and visualization results to analyze category-level behavior more completely.

## 6. Conclusions

This paper presents an improved YOLO-based detection model for potato leaf disease images. The study begins from the practical difficulties of potato disease detection, including deep semantic weakening, field background interference, and multi-scale lesion texture variation. To address these problems, the model introduces DSE, LSA, and MSRA.

DSE strengthens deep feature extraction and helps the model preserve weak disease information. LSA improves attention to spatially distributed lesion regions. MSRA refines multi-scale texture and lesion-boundary information after SPPF. Together, these components form a disease-oriented detection framework for potato leaf images.

The experimental results show that the proposed model achieves 88.92% mAP50 and 83.51% AR, which are 7.61 and 5.66 percentage points higher than the YOLO26s baseline, respectively. The final model has 10.824 M parameters, 34.98 GFLOPs, and 118.6 FPS. These results indicate that the proposed framework can improve potato leaf disease detection accuracy while maintaining real-time performance, providing a useful reference for precision agriculture disease monitoring.

## Figures and Tables

**Table 1:** Training settings used in the experiments.

Item	Setting
Framework	YOLO26s implementation based on PyTorch 2.1
Optimizer	Adam optimizer implemented in PyTorch 2.1
Initial learning rate	$5\times 10^{-4}$
Final learning rate factor	$1\times 10^{-2}$
Momentum	0.9
Cosine learning rate	True
Warmup epochs	5
Epochs	100
Batch size	32
Input size	640 × 640
Workers	12
Device	GPU 0
Mosaic/close mosaic	1.0/last 10 epochs
Auto augmentation	RandAugment
Random erasing	0.4
HSV augmentation	0.015/0.7/0.4
Translation/scaling	0.1/0.5
Horizontal/vertical flip	0.5/0.0
Mixup/CutMix/shear/perspective	0.0/0.0/0.0/0.0

**Table 2:** Overall performance comparison between the baseline and proposed model.

Model	mAP50 (%)	AR (%)	Params (M)	GFLOPs (G)	FPS
YOLO26s baseline	81.31	77.85	10.010	22.84	156.9
Proposed model	88.92	83.51	10.824	34.98	118.6

**Table 3:** Performance comparison of different detection models on the potato leaf disease dataset.

Model	mAP50 (%)	AR (%)	Params (M)	GFLOPs (G)	FPS
YOLOv5S	80.75	75.79	9.11	23.84	252
YOLOv8S	81.02	75.81	11.13	28.45	272
YOLOv11S	81.26	76.45	9.41	21.31	184
YOLO26s	81.31	77.85	10.01	20.53	142
RTDETR_L	77.21	75.73	31.99	103.45	53
SPPELAN	80.75	73.62	15.77	20.14	124
EfficientDet-D0	81.24	72.03	3.83	6.71	61
Proposed model	88.92	83.51	10.824	34.98	118.6

**Table 4:** Comparison of attention mechanisms based on the YOLO26s baseline.

Model	mAP50 (%)	AR (%)	Params (M)	GFLOPs (G)	FPS
Baseline	81.31	77.85	10.010	22.84	156.9
Baseline + CA	82.77	80.35	10.016	22.85	183.3
Baseline + DCAFE	83.66	79.59	10.023	22.87	173.4
Baseline + EMA	84.06	80.35	10.010	22.91	176.0
Baseline + LSKA	85.79	80.44	10.081	23.06	186.6
Baseline + SimAM	84.44	79.32	10.010	22.84	182.4

**Table 5:** Backbone enhancement position experiment.

Model	mAP50 (%)	AR (%)	Params (M)	GFLOPs (G)	FPS
Baseline	81.31	77.85	10.010	22.84	156.9
BB34-P5	85.48	78.50	11.355	38.61	87.9
BB3-P5	85.16	79.04	10.301	26.44	122.6
BB4-P5	86.37	80.73	11.064	35.01	124.1
Full-BB-P5	83.51	78.26	11.413	40.05	62.8

**Table 6:** Effect of the number of Mona adapters.

Model	mAP50 (%)	AR (%)	Params (M)	GFLOPs (G)	FPS
Baseline	81.31	77.85	10.010	22.84	156.9
Mona N = 1	85.21	78.51	9.655	22.55	185.3
Mona N = 2	86.11	80.38	9.699	22.59	174.5
Mona N = 3	85.80	77.95	9.743	22.62	163.3
Mona N = 4	84.77	77.56	9.786	22.66	156.9

**Table 7:** Ablation experiment of the proposed modules.

DSE	LSA	MSRA	mAP50	AR	Params	GFLOPs	FPS
✓	–	–	86.37	80.73	11.064	35.01	126.4
–	✓	–	85.79	80.44	10.081	23.06	186.2
–	–	✓	86.11	80.38	9.699	22.59	178.4
✓	✓	–	87.21	81.94	11.135	35.23	123.7
✓	–	✓	87.94	82.66	10.753	34.76	119.5
–	✓	✓	86.93	81.28	9.770	22.81	173.5
✓	✓	✓	88.92	83.51	10.824	34.98	118.6

**Table 8:** Complexity and speed comparison of representative detectors.

Model	Params (M)	GFLOPs (G)	FPS
YOLOv5S	9.11	23.84	252
YOLOv8S	11.13	28.45	272
YOLOv9S	7.17	26.74	101
YOLOv11S	9.41	21.31	184
YOLO26s	10.01	20.53	142
RTDETR_L	31.99	103.45	53
SPPELAN	15.77	20.14	124
EfficientDet-D0	3.83	6.71	61
Proposed model	10.824	34.98	118.6

## Data Availability

The original contributions presented in this study are included in the article. Further inquiries can be directed to the corresponding authors.

## Abbreviations

The following abbreviations are used in this manuscript:

AP	Average precision
FPS	Frames per second
FLOPs	Floating-point operations
IoU	Intersection over union
LSKA	Large separable kernel attention
mAP	Mean average precision
NMS	Non-maximum suppression
SPPF	Spatial pyramid pooling fast
YOLO	You only look once
